# An Integrated Algorithmic MADM Approach for Heart Diseases’ Diagnosis Based on Neutrosophic Hypersoft Set with Possibility Degree-Based Setting

**DOI:** 10.3390/life12050729

**Published:** 2022-05-13

**Authors:** Atiqe Ur Rahman, Muhammad Saeed, Mazin Abed Mohammed, Sujatha Krishnamoorthy, Seifedine Kadry, Fatma Eid

**Affiliations:** 1 Department of Mathematics, University of Management and Technology, Lahore 54000, Pakistan; aurkhb@gmail.com (A.U.R.); muhammad.saeed@umt.edu.pk (M.S.); 2 College of Computer Science and Information Technology, University of Anbar, Anbar 31001, Iraq; mazinalshujeary@uoanbar.edu.iq; 3 Zhejiang Bioinformatics International Science and Technology Cooperation Center, Wenzhou-Kean University, Wenzhou 325060, China; 4 Wenzhou Municipal Key Lab of Applied Biomedical and Biopharmaceutical Informatics, Wenzhou-Kean University, Wenzhou 325060, China; 5 Department of Applied Data Science, Noroff University College, 4612 Kristiansand, Norway; skadry@gmail.com; 6 Technology Management, College of Business, Stony Brook University, Stony Brook, NY 11794, USA; fatma.eid@stonybrook.edu

**Keywords:** hypersoft set, neutrosophic hypersoft set, possibility neutrosophic hypersoft set, decision-making, Cleveland data set

## Abstract

The possibility neutrosophic hypersoft set (pNHs-set) is a generalized version of the possibility neutrosophic soft set (pNs-set). It tackles the limitations of the pNs-set regarding the use of the multi-argument approximate function. This function maps sub-parametric tuples to a power set of the universe. It emphasizes the partitioning of each attribute into its respective attribute-valued set. These features make it a completely new mathematical tool for solving problems dealing with uncertainties. This makes the decision-making process more flexible and reliable. In this study, after characterizing some elementary notions and algebraic operations of the pNHs-set, Sanchez’s method (a classical approach for medical diagnosis) is modified under the pNHs-set environment. A modified algorithm is proposed for the medical diagnosis of heart diseases by integrating the concept of the pNHs-set and the modified Sanchez’s method. The authenticity of the proposed algorithm is evaluated through its implementation in a real-world scenario with real data from the Cleveland data set for heart diseases. The beneficial aspects of the proposed approach are evaluated through a structural comparison with some pertinent existing approaches.

## 1. Introduction

The fuzzy set (F-set) [1] and intuitionistic fuzzy set (IF-set) [2] are considered pertinent mathematical approaches to undertake numerous complicated problems concerning diverse uncertainties, in different scientific disciplines. The first one highlights the grade of the true belongingness of a certain entity from the initial sample space, whereas the second one puts emphasis on the grade of true membership and the grade of nonmembership with the provision of their dependency on each other. These theories portray some sort of scantiness concerning the stipulation of due status of the grade of indeterminacy. Such obstruction is dealt with by the development of the neutrosophic set (N-set) [3], which not only reflects the due status of the grade of indeterminacy, but also puts aside the condition of dependency.

The F-set, IF-set, and N-set have some sort of complications that keep them from resolving problems linked with uncertainties. The motive for these impediments is, perhaps, the meagerness of the parameterization mode. It calls for a mathematical device free of all such hurdles to undertake such concerns. This meagerness is determined with the characterization of the soft set (S-set) [4], which is an innovative parameterized collection of subsets of a universal set. The researchers Maji et al. [5] and Ali et al. [6] studied various basic properties, operations, laws, relations, and functions of the S-set with applications in decision-making. In order to equip the F-set, IF-set, and N-set with parameterization tools, their hybridized structures, the fuzzy soft set (Fs-set) [7], intuitionistic fuzzy soft set (IFs-set) [8], and neutrosophic soft set (Ns-set) [9], respectively, with the S-set have been conceptualized.

In numerous real-life situations, distinctive attributes necessarily need to be classified into parametric non-overlapping sets, but the S-set is not enough to manage such sets. Hypersoft set theory (Hs-set) [10] is projected to make the S-set compatible with such settings. The Hs-set is an extension of the S-set, as it changes the soft approximate function into a multi-argument function (maa-function). Several basic properties, aggregation operations, laws, relations, and functions of the Hs-set were explored by Abbas et al. [11] and Saeed et al. [12] for proper understanding and further employment in various fields. Rahman et al. [13] discussed the hybrids of the Hs-set with F-set and IF-set and N-set under a complex set environment. They [14] developed the notions of convexity and concavity under the Hs-set environment. Debnath [15] applied the fuzzy Hs-set (FHs-set) to decision-making by using its weight operator. Yolcu et al. [16] developed the intuitionistic fuzzy hypersoft set (IFHs-set) and discussed its various operations. Saqlain et al. [17] investigated certain properties, aggregation operations, similarity measures, and the accuracy function of the neutrosophic Hs-set (NHs-set). They employed decision-making techniques such as TOPSIS to deal with real-world problems using the accuracy function. Saeed et al. [18,19] introduced the concept of mappings under the NHs-set environment and applied them in decision-making for medical diagnosis. They [20,21] also discussed the representation of the NHs-set in graphs along with certain operations and products with application in decision-making. Rahman et al. [22,23] investigated the parameterization of the NHs-set with fuzzy, intuitionistic fuzzy, and neutrosophic settings with applications for decision support systems.

The research gap, novelty, and motivation of the proposed study can be viewed from the following points:

1. The approaches such as the Fs-set, IFs-set, Ns-set, etc., have been extensively utilized in various circumstances to resolve decision-making problems. Nevertheless, under several conditions, these structures demonstrate inadequacies in categorizing the entities according to their possibility grades. In other words, it can be interpreted that in the existing literature, the possibility degree of each element is regarded as one. However, in several realistic applications, different individuals may assign different possibility grades to each entity. To handle such concerns, Alkhazaleh et al. [24] explored the possibility Fs-set (pFs-set), which ensures the allocation of a possibility grade with every approximate element in the fs-set. However, such a model is not compatible with the use of the nonmembership grade. In order to tackle it and address it more appropriately, Bashir et al. [25] introduced the possibility IFs-set (pIFs-set). In the pIFs-set, the ifs numbers are assessed through the use of the possibility grade while computing the ranking analysis, but the degree of indeterminacy is ignored. This shortcoming was addressed by developing the possibility Ns-set (pNs-set) in [26].
2. While in real-world observations, we come across various situations when the parameters are not enough to make the right decision, they demand being classified into their respective parametric-valued-based sets. Such situations have been tackled with the development of Hs-sets, which employ a particular mapping maa-function to manage these settings.
3. The nHs-set is the generalization of the Fs-set, IFs-set, Ns-set, FHs-set, and IFHs-set. Since these models have limitations such as the degree of indeterminacy and the maa-function being ignored in the fs-set and ifs-set, the maa-function being ignored in the Ns-set, and the degree of indeterminacy being ignored in the FHs-set and IFHs-set, the NHs-set is meant to tackle such limitations.
4. The proposed model, the possibility neutrosophic hypersoft set (pNHs-set), is a novel structure, which not only generalizes the existing models, but also makes them adequate with the use of the maa-function. In the pNHs-set, a possibility degree is attached to the neutrosophic numbers of the maa-function of the pNHs-set to judge their uncertain nature. In this sense, it is a more flexible and generalized model to deal with uncertain data diligently.

The following points describe the major contributions of this research:

1. Some elementary notions and algebraic operations of the pNHs-set are characterized with the support of explicatory examples.
2. In contrast with existing approaches, the attributes and sub-attributive values (real data) of the Cleveland data set for the diagnosis of heart diseases are first analyzed on the basis of their operational and linguistic roles, and then, their linguistic values are transformed into possibility grades by using a suitable algebraic approach.
3. Sanchez’s method (a classical approach for medical diagnosis) is modified under the pNHs-set environment to establish a relationship among patients under observation, prescribed attributes, and the decision-makers (medical specialists).
4. A modified algorithm is put forward for the medical diagnosis of heart diseases by integrating the theory of pNHs-sets, the prescribed real data of Cleveland data set, and the modified Sanchez’s method.
5. The validity of the proposed algorithm is assessed by its implementation in a real-world problem-based scenario.
6. The advantageous aspects of the proposed approach are judged through a structural comparison with some relevant existing approaches.

For proper understanding of the concept, all the abbreviations used are explained in Table 1.

## 2. Preliminaries

This portion of the paper presents some elementary terms and definitions by reviewing the existing literature for a vivid understanding of the proposed study.

**Definition** **1** ([10]). *An*Hs-set*over Z is a set of pairs (W,H), where H is the Cartesian product of $H^{i},i=1,2,3,\ldots ,n,H^{i}\cap H^{j}=\emptyset$ for all i≠j having attribute values of attributes ${\hat{a}}^{i},i=1,2,3,\ldots ,n,{\hat{a}}^{i}\neq {\hat{a}}^{j},i\neq j$, respectively and W:H→P(Z). An Hs-set (W,H) can also be written as $Z=\left(W,H\right)=\{\left({\hat{h}}_{1},W\left({\hat{h}}_{1}\right)\right),\left({\hat{h}}_{2},W\left({\hat{h}}_{2}\right)\right),\left({\hat{h}}_{3},W\left({\hat{h}}_{3}\right)\right),\ldots ,\left({\hat{h}}_{k},W\left({\hat{h}}_{k}\right)\right)\}$, where k is the cardinality (number of elements) in the set H. All $W({\hat{h}}_{\alpha }),\alpha =1,2,3,\ldots ,k$ are the subsets of Z and are known as multi-argument approximate elements of the Hs-set (W,H). In other words, an Hs-set (W,H) can be viewed as a parameterized collection of the elements of the initial universe Z.*

**Definition** **2** ([10]). *An Hs-set (W,H) is called a fuzzy Hs-set, intuitionistic Hs-set, and neutrosophic Hs-set if P(Z) in W:H→P(Z) is replaced with F(Z), IF(Z) and N(Z), respectively, where F(Z), IF(Z) and N(Z) are families of all fuzzy, intuitionistic fuzzy, and neutrosophic subsets on Z, respectively.*

**Definition** **3** ([26]). *A pNs-set $R_{S}$ is defined as*

$$R_{S}=\left\{\begin{array}{c} \left(\zeta_{S}\left(\hat{a}\right),\psi_{S}\left(\hat{a}\right)\right);\zeta_{S}\left(\hat{a}\right)\in N\left(Z\right),\psi_{S}\left(\hat{a}\right)\in F\left(Z\right)\;\;and\;\;\hat{a}\in A \end{array}\right\}$$

*where A⊆E (a set of parameters), $\zeta_{S}:A\to N\left(Z\right)$, and $\psi_{S}:A\to F\left(Z\right)$.*

## 3. Possibility Neutrosophic Hypersoft Set and Set-Theoretic Operations

This section presents the definition and set-theoretic operations of the pNHs-set with numerical examples. We first discuss the real-world scenario, which demands the setting of the pNHs-set. It is a matter of common observation that in any recruitment process, a panel is constituted to interview the initially scrutinized candidates. This panel usually consists of a chairperson and other members having expertise in the relevant field. All members of the panel are directed to assess the capability and suitability of the candidates for the advertised posts by considering pre-set evaluating parameters and their sub-parametric values in the form of sets. They are further directed to provide their expert opinions in three dimensions, i.e., they may recommend, reject, or be neutral regarding the assessment of candidates corresponding to multi-argument tuples. The chairperson is empowered to analyze the expert opinions of the decision-makers in accordance with their level of acceptance. In short, there are three situations in this scenario that must be tackled in one model:

1. The situation that declares the essential classification of the parameters into their related sub-parametric values in the form of different sets.
2. The situation that requires the claim of the maa-function that it is proficient to undertake the multi-argument domain in the form of sub-parametric-valued tuples.
3. The situation that compels the decision-makers to give their expert judgments in the form of neutrosophic values, which guarantee the three dimensions, i.e., truth (real membership), indeterminacy (neutral value), and falsity (real nonmembership), of the opinion of decision-makers.
4. The situation that requires the reflection of the possibility degree to evaluate the approval level of the collected expert judgments for the objects being considered.

The exiting literature is inadequate to provide any mathematical model to tackle all the above-mentioned situations collectively in one model. This shortcoming leads to the motivation of this study. The proposed model, the pNHs-set, is capable of managing all the above-described situations collectively as a single structure. The pNHs-set has three parts: (i) hypersoft setting, (ii) neutrosophic setting, and (iii) possibility-degree-based setting. The pNHs-set manages Situations (i)–(ii) by employing hypersoft setting; Situation (iii) is tackled by using the neutrosophic setting; the last Situation (iii) is managed by using the possibility-degree based setting. There are many other real-world scenarios such as product selection, medical diagnosis, project selection, risk analysis, etc., that require the pNHs setting.

**Definition** **4.** *A pNHs-set $F_{\psi }$ over hypersoft universe (Z,J) is stated by the pairs $F_{\psi }=\left\{\begin{array}{c} \left(\delta ,\left\{\left(\frac{{\hat{z}}_{1}}{F\left(\delta \right)\left(\hat{z}\right)},\psi \left(\delta \right)\left(\hat{z}\right)\right):\hat{z}\in Z\right\}\right):\delta \in J \end{array}\right\}$ where $J_{i}$ are non-overlapping parametric sets for parameters $a_{i},i=1,2,\ldots ,n$, respectively, such that $J=J_{1}\times J_{2}\times \cdots \times J_{n}$, $F_{\psi }:J\to N_{Z}\times I_{Z}$, $F:J\to N_{Z}$, $\psi :J\to I_{Z}$,   $I_{Z}\in F\left(Z\right)$, and $N_{Z}\in N\left(Z\right)$, respectively; $F\left(\delta \right)\left(\hat{z}\right)$ is a neutrosophic number of $\hat{z}\in Z$ in F(δ), and $\psi \left(\delta \right)\left(\hat{z}\right)$ is a possibility grade of $\hat{z}\in Z$ in F(δ). Therefore, $F_{\psi }\left(\delta_{i}\right)$ can be stated as:*

$$F_{\psi }\left(\delta_{i}\right)=\left\{\begin{array}{c} \left(\frac{{\hat{z}}_{1}}{F\left(\delta_{i}\right)\left({\hat{z}}_{1}\right)},\psi \left(\delta_{i}\right)\left({\hat{z}}_{1}\right)\right),\left(\frac{{\hat{z}}_{2}}{F\left(\delta_{i}\right)\left({\hat{z}}_{2}\right)},\psi \left(\delta_{i}\right)\left({\hat{z}}_{2}\right)\right),\ldots ,\left(\frac{{\hat{z}}_{n}}{F\left(\delta_{i}\right)\left({\hat{z}}_{n}\right)},\psi \left(\delta_{i}\right)\left({\hat{z}}_{n}\right)\right) \end{array}\right\}$$

*Note: for convenience, the pNHs-set is denoted by $F_{\psi }$ and its family is represented by $\Omega_{pnhss}$.*

**Example** **1.** *Suppose the medical superintendent of a civil hospital creates a committee consisting of heart specialists to assess the heart diseases by observing appropriate parameters and their relevant sub-parametric values for the sake of research. The committee is headed by a chairperson, who is responsible for the final decision. Other members of the committee will provide their expert opinions as decision-makers, and the chairman has been empowered to scrutinize the received opinions in accordance with their acceptance level. Four types of heart diseases (alternatives) are taken into considerations, which are enclosed in the set of discourse $Z=\{{\hat{D}}_{1},{\hat{D}}_{2},{\hat{D}}_{3},{\hat{D}}_{4}\}$. The members of the committee have set the parameters $a_{1}$ = chest pain type, $a_{2}$ = resting blood pressure (mmHg), and $a_{3}$ = serum cholesterol (mg/dL) with their mutual consensus. After keen observation, the parameters are further classified into their related parametric-valued sets, which are $J_{1}=\left\{a_{11}=typicalangina,a_{12}=atypicalangina\right\}$, $J_{2}=\left\{a_{21}=150,a_{22}=180\right\}$, and $J_{3}=\left\{a_{31}=320\right\}$, respectively. In order to obtain the parametric tuples, their Cartesian product is computed as $J=J_{1}\times J_{2}\times J_{3}=\left\{\delta_{1},\delta_{2},\delta_{3},\delta_{4}\right\}$. By keeping in view the preference of the parametric tuples, the members of the committee are directed to provide their opinions in the form neutrosophic components, i.e., membership grade, indeterminate grade, and nonmembership grade, for each disease. The expert opinions of the members and the possibility degree assigned by the chairperson for the acceptance level of the received opinions are collected as multi-argument approximate elements of the pNHs-set, which are given below:*

$$F_{\psi }\left(\delta_{1}\right)=\left\{\begin{array}{c} \left(\frac{{\hat{D}}_{1}}{≺0.3,0.1,0.2≻},0.2\right),\left(\frac{{\hat{D}}_{2}}{≺0.4,0.2,0.3≻},0.3\right),\left(\frac{{\hat{D}}_{3}}{≺0.5,0.3,0.4≻},0.4\right),\left(\frac{{\hat{D}}_{4}}{≺0.6,0.4,0.5≻},0.5\right) \end{array}\right\}$$

$$F_{\psi }\left(\delta_{2}\right)=\left\{\begin{array}{c} \left(\frac{{\hat{D}}_{1}}{≺0.7,0.2,0.3≻},0.8\right),\left(\frac{{\hat{D}}_{2}}{≺0.6,0.3,0.4≻},0.8\right),\left(\frac{{\hat{D}}_{3}}{≺0.6,0.4,0.5≻},0.7\right),\left(\frac{{\hat{D}}_{4}}{≺0.5,0.5,0.6≻},0.6\right) \end{array}\right\}$$

$$F_{\psi }\left(\delta_{3}\right)=\left\{\begin{array}{c} \left(\frac{{\hat{D}}_{1}}{≺0.5,0.1,0.1≻},0.1\right),\left(\frac{{\hat{D}}_{2}}{≺0.4,0.1,0.2≻},0.2\right),\left(\frac{{\hat{D}}_{3}}{≺0.5,0.1,0.3≻},0.3\right),\left(\frac{{\hat{D}}_{4}}{≺0.6,0.2,0.4≻},0.4\right) \end{array}\right\}$$

$$F_{\psi }\left(\delta_{4}\right)=\left\{\begin{array}{c} \left(\frac{{\hat{D}}_{1}}{≺0.7,0.1,0.2≻},0.2\right),\left(\frac{{\hat{D}}_{2}}{≺0.5,0.1,0.3≻},0.3\right),\left(\frac{{\hat{D}}_{3}}{≺0.6,0.4,0.4≻},0.4\right),\left(\frac{{\hat{D}}_{4}}{≺0.7,0.2,0.5≻},0.5\right) \end{array}\right\}$$

*Then, $F_{\psi }$ is a pNHs-set over (Z,J). Its matrix representation is:*

$$F_{\psi }=\left(\begin{array}{cccc} \left(≺0.3,0.1,0.2≻,0.2\right) & \left(≺0.4,0.2,0.3≻,0.3\right) & \left(≺0.5,0.3,0.4≻,0.4\right) & \left(≺0.6,0.4,0.5≻,0.5\right)\\ \left(≺0.7,0.2,0.3≻,0.8\right) & \left(≺0.6,0.3,0.4≻,0.8\right) & \left(≺0.6,0.4,0.5≻,0.7\right) & \left(≺0.5,0.5,0.6≻,0.6\right)\\ \left(≺0.5,0.1,0.1≻,0.1\right) & \left(≺0.4,0.1,0.2≻,0.2\right) & \left(≺0.5,0.1,0.3≻,0.3\right) & \left(≺0.6,0.2,0.4≻,0.4\right)\\ \left(≺0.7,0.1,0.2≻,0.2\right) & \left(≺0.5,0.1,0.3≻,0.3\right) & \left(≺0.6,0.4,0.4≻,0.4\right) & \left(≺0.7,0.2,0.5≻,0.5\right) \end{array}\right).$$

*In this pNHs-set, the first element (≺0.3,0.1,0.2≻,0.2) states that all the decision-makers have provided collectively 0.3 (30%) as the membership grade, 0.1(10%) as the indeterminate grade, and 0.2 (20%) as the nonmembership grade for disease ${\hat{D}}_{1}$, and 0.2 (20%) is the possibility degree assigned by the chairperson for the acceptance level of expert opinions ≺0.3,0.1,0.2≻ to ${\hat{D}}_{1}$ by keeping in view the parametric tuple $\delta_{1}$. Similarly, all other approximate elements and their values are computed in the same manner.*

**Definition** **5.** *Let $A_{\psi },B_{\zeta }\in \Omega_{pnhss}$, then:*

(i) *$A_{\psi }\cup B_{\zeta }$ is a pNHs-set $C_{\nu }$ with C(δ)=⊔{A(δ),B(δ)}, and ν(δ)=max{ψ(δ),ζ(δ)}.*
(ii) *$A_{\psi }\cap B_{\zeta }$ is also a pNHs-set $D_{\omega }$ with D(δ)=⊓{A(δ),B(δ)}, and ω(δ)=min{ψ(δ),ζ(δ)}.*

*Here, ⊔ and ⊓ denote the neutrosophic union and neutrosophic intersection, respectively.*

**Example** **2.** *Assuming the data from Example 1, two pNHs-sets $A_{\psi },B_{\zeta }\in \Omega_{pnhss}$ are constructed whose matrix notations are provided as*

$$A_{\psi }=\left(\begin{array}{cccc} \left(≺0.1,0.2,0.3≻,0.2\right) & \left(≺0.2,0.3,0.4≻,0.3\right) & \left(≺0.3,0.4,0.5≻,0.4\right) & \left(≺0.4,0.5,0.6≻,0.5\right)\\ \left(≺0.5,0.5,0.6≻,0.8\right) & \left(≺0.6,0.4,0.5≻,0.8\right) & \left(≺0.7,0.3,0.4≻,0.7\right) & \left(≺0.9,0.1,0.2≻,0.6\right)\\ \left(≺0.4,0.3,0.4≻,0.1\right) & \left(≺0.6,0.4,0.5≻,0.2\right) & \left(≺0.7,0.2,0.3≻,0.3\right) & \left(≺0.4,0.1,0.2≻,0.4\right)\\ \left(≺0.6,0.2,0.3≻,0.2\right) & \left(≺0.7,0.3,0.4≻,0.3\right) & \left(≺0.5,0.2,0.3≻,0.4\right) & \left(≺0.7,0.2,0.3≻,0.5\right) \end{array}\right)$$

*and*

$$B_{\zeta }=\left(\begin{array}{cccc} \left(≺0.2,0.1,0.2≻,0.3\right) & \left(≺0.3,0.2,0.3≻,0.4\right) & \left(≺0.4,0.3,0.4≻,0.5\right) & \left(≺0.5,0.4,0.5≻,0.6\right)\\ \left(≺0.6,0.4,0.5≻,0.9\right) & \left(≺0.7,0.3,0.4≻,0.9\right) & \left(≺0.8,0.2,0.3≻,0.8\right) & \left(≺1.0,0.0,0.1≻,0.7\right)\\ \left(≺0.5,0.2,0.3≻,0.2\right) & \left(≺0.7,0.3,0.4≻,0.3\right) & \left(≺0.8,0.1,0.2≻,0.4\right) & \left(≺0.5,0.0,0.1≻,0.5\right)\\ \left(≺0.7,0.1,0.2≻,0.3\right) & \left(≺0.8,0.2,0.3≻,0.4\right) & \left(≺0.6,0.1,0.2≻,0.5\right) & \left(≺0.8,0.1,0.2≻,0.6\right) \end{array}\right)$$

*then $C_{\nu }=A_{\psi }\cup B_{\zeta }=B_{\zeta }$ and $D_{\omega }=A_{\psi }\cap B_{\zeta }=A_{\psi }$.*

## 4. Proposed Methodology and Algorithmic Implementation

**Figure 1:**
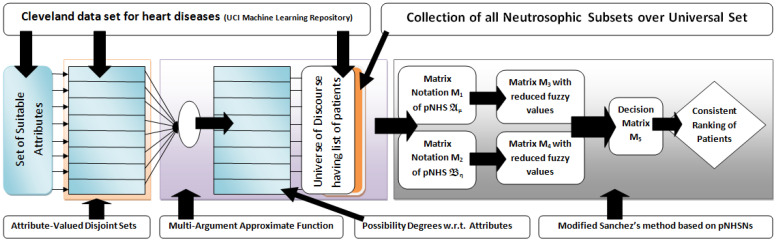
Adopted methodology of the paper.

In this section, the medical diagnosis method of [27] is employed with partial modifications to diagnose heart diseases using real data of the Cleveland data set [28] under the pNHs-set environment. The pictorial representation of the complete adopted methodology of the paper is presented in Figure 1.

### 4.1. Modified Sanchez’s Method

Sanchez’s method [27] is a classical technique that is used to relate the parameters, universal sets, and the opinions of decision-makers in one model. It employs the concept of matrix theory to develop such a relationship. It is usually used for medical diagnosis, but can be applied in any other scenario with partial modifications. It requires two main matrices, which are constructed from parameters, universal sets, and the opinions of decision-makers transitively. Now, we present this technique with modifications for our proposed model.

Let $U=\{{\hat{p}}_{1},{\hat{p}}_{2},\ldots ,{\hat{p}}_{m}\}$ be the initial universe having a list of patients and $G=G_{1}\times G_{2}\times \cdots \times G_{n}=\left\{{\hat{g}}_{1},{\hat{g}}_{2},\ldots ,{\hat{g}}_{n}\right\}$ be the collection of attribute-valued tuples where $G_{i}$ are non-overlapping attribute-valued sets with respect to prescribed attributes ${\hat{e}}_{i},i=1,2,\ldots ,n$ of the Cleveland data set. Let a group of decision-makers $\hat{D}=\left\{{\hat{D}}_{1},{\hat{D}}_{2},{\hat{D}}_{3},\ldots ,{\hat{D}}_{k}\right\}$ participate in the diagnosis process. For two pNHs-sets $A_{\psi },B_{\zeta }\in \Omega_{pnhss}$, we have their respective matrix notations $M_{1}={\left[a_{ij}\right]}_{m\times n}$ and $M_{2}={\left[b_{ij}\right]}_{n\times k}$, respectively. The matrix $M_{1}$ is a U−G matrix having fuzzy values corresponding to attribute-valued tuples in G as entries, whereas $M_{2}$ is a $G-\hat{D}$ matrix having pNHs values assigned by decision-makers to attribute-valued tuples in G as entries. After transforming pNHs values to reduced fuzzy values, the matrices $M_{1}$ and $M_{2}$ are transformed to $M_{3}={\left[c_{ij}\right]}_{m\times n}$ and $M_{4}={\left[d_{ij}\right]}_{n\times k}$, respectively. The decision matrix $M_{5}={\left[e_{ij}\right]}_{m\times k}$ is obtained by taking ⨂ (ordinary product of matrices) of $M_{3}$ and $M_{4}$.

### 4.2. Cleveland Data Set

The Cleveland data set [28] is meant for the diagnostic study of heart diseases. In this data set, 303 patients were observed for the diagnosis of heart diseases by considering 76 attributes (however, 14 of them can be used for experiments and analysis) having five outcomes. The description of the 14 attributes is given in Table 2. Keeping in mind the further partitioning of attributes into their corresponding attribute-valued disjoint sets, six patients are chosen to be diagnosed for heart diseases by considering nine the most suitable attributes. As the proposed structure pNHs-set demands the further classification of parameters into their relevant sub-parametric values in the form of disjoint sets, in order to meet this demand, out of 14 parameters, only those 9 parameters were chosen, which have sub-parametric values within the Cleveland data set. As far as the choice of six patients is concerned, this was done to avoid the complexity of computations because the adopted approach Sanchez’s method is based on matrices and the consideration of more patients may lead to such complexity. However, the proper computer programming may be adopted to resolve such complexity. The descriptions of these nine attributes along with their prescribed values (data set) are provided in Table 3.

### 4.3. Operational Role of Selected Attributes

**Figure 2:**
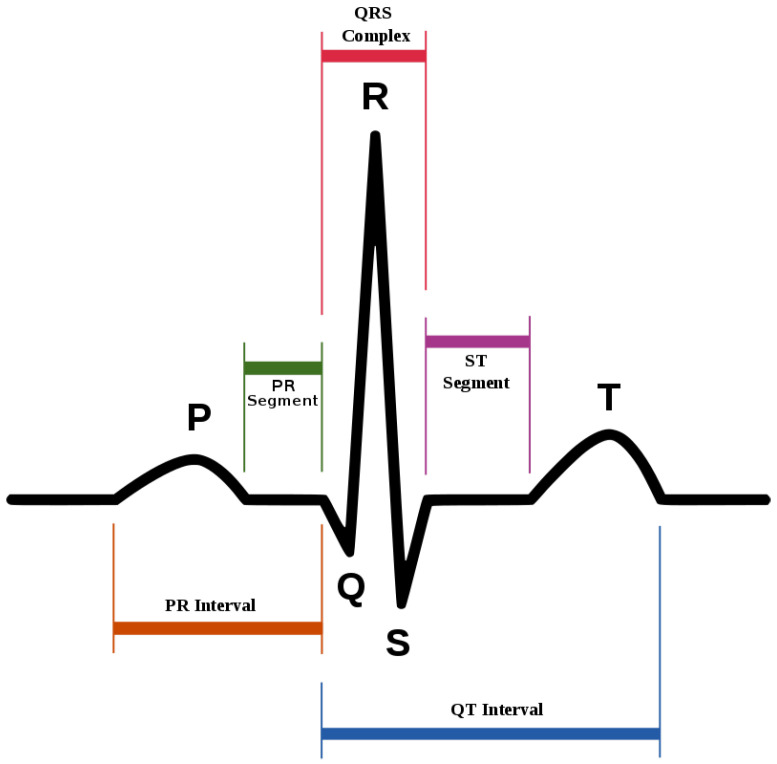
ST-segment in ECG (source: Wikipedia).

**Figure 3:**
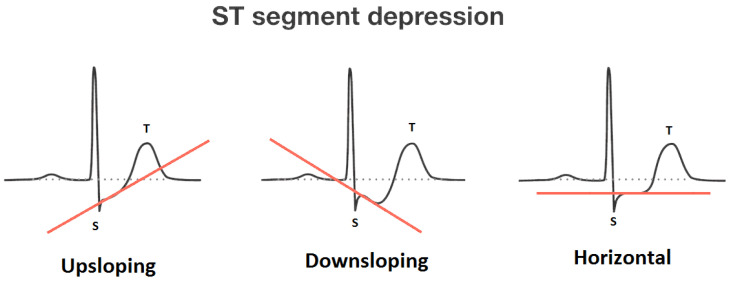
Sloping of the ST-segment (source: https://litfl.com/st-segment-ecg-library (accessed on 3 October 2021)).

In this part of the section, the operational role of the selected attributes is discussed to justify their selection for diagnosing heart diseases:

1. **Age**: Aging is an independent risk factor for heart diseases. Although the risk of heart disease is higher in older people (60 years or more), with the association of certain other factors, younger people can be at risk. Medical specialists have categorized aging into four categories: up to 20 years, up to 40 years, up to 60 years, and above 60 years.
2. **Chest pain type**: Chest pain is perhaps the most well-known reason that individuals visit the trauma center. It changes relying on the individual. It likewise differs in: quality, force, span, area. It might feel like a sharp, agonizing feeling or a dull pain. It could be an indication of a genuine heart-related issue. Numerous normal causes that are not dangerous may likewise have caused it. Heart-related chest pain can be categorized into typical angina (TA), atypical angina (ATA), non-anginal pain, and asymptomatic (AM). Typical angina consists of (1) substernal chest pain or discomfort that is (2) provoked by exertion or emotional stress and (3) relieved by rest or nitroglycerine (or both). Atypical (probable) angina chest pain applies when two out of three criteria of classic angina are present. Non-anginal pain is applied to hospitalized patients in order to designate that they neither have an acute coronary syndrome nor display evidence of a coronary ischemia. Asymptomatic means there are no symptoms for the disease under consideration.
3. **Resting blood pressure**: Blood pressure is a particular kind of pressure that is exerted by the blood against the artery walls. Such pressure is further categorized as systolic and diastolic. The first one is produced when the heart drains off the blood into the blood vessels, and the second one is produced inside the arteries due to the relaxing state of the heart. Hypertension is the point at which the power of the blood is excessively high during heart compression or relaxing inside the arteries. The arteries may have an increased resistance against the flow of blood. Both pressures are measured in mm Hg. Typical blood pressure is systolic if under 120 and diastolic if under 80 (120/80). Raised blood pressure is systolic if 120 to 129 and diastolic under 80.
4. **Serum cholesterol**: Cholesterol is a sort of fat. It is also known as a lipid. It goes through our bloodstream in small particles wrapped inside proteins. These bundles are known as lipoproteins. LDL is one of the principal kinds of lipoproteins in our blood. The other principal type is high-thickness lipoproteins (HDLs). A third kind of lipid, called a triglyceride, likewise courses in our blood. Estimating our LDL (“bad” cholesterol), HDL (“good” cholesterol), and triglycerides will give us a number called our complete blood cholesterol, or serum cholesterol. Our body needs cholesterol to build healthy cells, but high levels of cholesterol can increase our risk of heart disease. With high cholesterol, we can develop fatty deposits in our blood vessels. Eventually, these deposits grow, making it difficult for enough blood to flow through our arteries. Serum cholesterol levels is calculated by adding HDL and LDL cholesterol levels with 20 percent of triglycerides. It ranges from 126 mg/dL to 564 mg/dL. The ranges of certain cholesterol are given in Table 4.
5. **Fasting blood sugar**: A large number of people who suffer a heart disease have high glucose due to the “stress response”. This means that even people who are not diabetic may have high blood sugar. Its ranges are given in Table 5. Its normal range is 120 mg/dL for a healthy person.
6. **Maximum heart rate achieved**: Heart rate is a major determinant of oxygen consumption in patients with ischemic heart disease. Its maximum value that can be achieved ranges from 71 beats per minute to 195 beats per minute.
7. **Oldpeak and slope**: Oldpeak: ST (S = shock, T = toxicity) depression induced by exercise relative to rest is considered a reliable electrocardiogram (ECG) finding for the diagnosis of obstructive coronary disorders. It is measured in mm and ranges from 0.0 to 0.5. Its graphical representation is given in Figure 2. The slope of the peak exercise ST-segment has three categories: upsloping, flat (horizontal) and downsloping. The graphical depiction of ST-sloping is provided in Figure 3.
8. **Thal**: This is a blood disorder known as thalassemia. It can be classified as null (value = 0), fixed defect (value = 3, no blood flow in some part of the heart), normal blood flow (value = 6), and reversible defect (value = 7; blood flow is observed, but it is not normal). Usually, the first category is ignored when diagnosing heart diseases.

### 4.4. Possibility Grades Corresponding to Selected Attributes

In this segment, the prescribed values (values assigned in the Cleveland data set) of the selected attributes are transformed into their respective possibility grades by adopting a suitable transformative criterion. The possibility grade corresponding to each attribute is obtained by dividing its prescribed value by its highest prescribed value. The maximum prescribed value for age is taken as 80 years, and the maximum values for other attributes are in accordance with the Cleveland data set. The possibility grades for all selected attributes are presented in Table 6.

### 4.5. Scenario and Statement of the Problem

Mathematical modeling for the clinical diagnosis of certain diseases has gained great interest of researchers. This modeling may involve real or hypothetical information/data. With the development of neutrosophic sets (N-sets), researchers have been attracted to neutrosophic modeling for medical diagnosis with uncertain scenarios. Many extensions and generalizations have been introduced to N-sets. One of them is the possibility neutrosophic hypersoft set (pNHs-set), which not only generalizes the existing models, but also addresses their inadequacies regarding coping with the further partitioning of attributes into attribute-valued disjoint sets and the use of possibility grades collectively. A few works have been reported in the literature regarding mathematical modeling for clinical diagnosis based on extensions of the fuzzy set and its parameterization with real data. This study employed a novel context of the pNHs-set for the clinical diagnosis of some patients regarding heart diseases using the real data of the Cleveland data set.

### 4.6. Proposed Algorithm and Implementation

In this section, an algorithm is proposed by using the concept of the aggregation of the pNHs-set for the diagnosis of heart diseases in patients. In multidisciplinary research, various kinds of software and programming languages are used, e.g., mathematicians like to use LATEX, MATLAB, MS WORD, etc., computer science researchers prefer to use python, C++ etc., and physicists and chemists prefer scientific workplace. Many converters are available online for the inter-conversion of these programs and coding. In order to increase the readability and understandability of the proposed study, it is pertinent to adopt an easy, but comprehensive way to describe the procedural flow of the proposed algorithm so that researchers may convert it according to their field of interest. As the scope of the proposed study is multidisciplinary areas of research, a simple procedure was adopted to explain the procedural flow of the proposed algorithm for the facilitation of the researchers (readers).

Now, the above algorithm is explained with the help of the following example.

**Figure 4:**
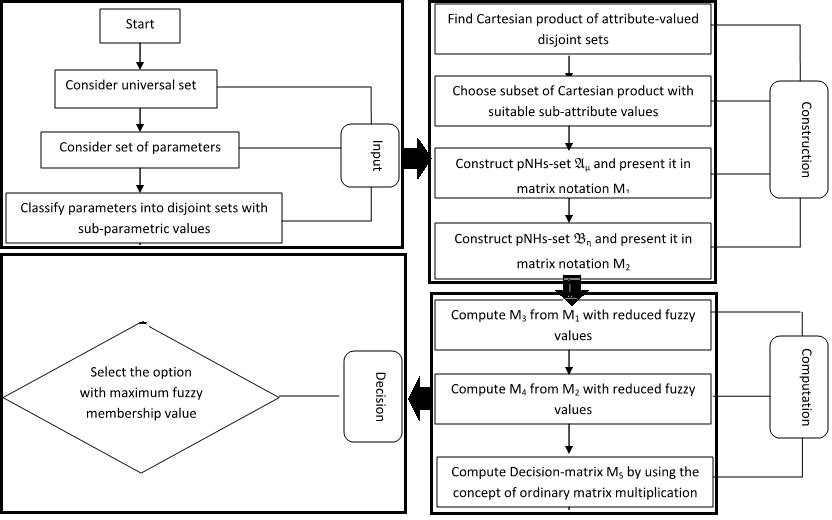
Flowchart of Algorithm 1.

The steps of Algorithm 1 are summarized as flow chart in Figure 4.
**Algorithm 1** Diagnosis of heart diseases through aggregation of the pNHs-set.▹**Start**▹**Input:**1. Consider U as the universe of discourse consisting of patients ${\hat{p}}_{1},{\hat{p}}_{2},{\hat{p}}_{3},\ldots ,{\hat{p}}_{k}$ under observation.2. Consider E as set of parameters ${\hat{e}}_{1},{\hat{e}}_{2},{\hat{e}}_{3},\ldots ,{\hat{e}}_{n}$.3. Classify n parameters into disjoint parametric-valued sets:$E^{1}=\left\{{\hat{e}}^{11},{\hat{e}}^{12},\ldots ,{\hat{e}}^{1n}\right\},\;E^{2}=\left\{{\hat{e}}^{21},{\hat{e}}^{22},\ldots ,{\hat{e}}^{2n}\right\},\;E^{3}=\left\{{\hat{e}}^{31},{\hat{e}}^{32},\ldots ,{\hat{e}}^{3n}\right\},\;...............................,\;...............................,\;E^{n}=\left\{{\hat{e}}^{n1},{\hat{e}}^{n2},\ldots ,{\hat{e}}^{nn}\right\},$▹. **Construction:**4. Determine $G=E^{1}\times E^{2}\times E^{3}\times \cdots \times E^{n}=\left\{{\hat{g}}_{1},{\hat{g}}_{2},{\hat{g}}_{3},\ldots ,{\hat{g}}_{r}\right\}$ with $r=\prod_{i=1}^{n}|E^{i}|$, where $|E^{i}|$ denotes the cardinality of sets $E^{i}$.5. Choose $H=\{{\hat{g}}_{1},{\hat{g}}_{2},{\hat{g}}_{3},\ldots ,{\hat{g}}_{s}\}$ a subset of G with s≤r in accordance with the priorities of attribute values.6. Construct pNHs-sets $A_{\psi }$ and $B_{\zeta }$, and represent in matrix notations $M_{1}={\left[a_{ij}\right]}_{m\times n}$ and $M_{2}={\left[b_{ij}\right]}_{n\times k}$, respectively.▹**Computation:**7. Compute $M_{3}={\left[c_{ij}\right]}_{m\times n}$, where $c_{ij}=\frac{|(T-I-F)+\psi |}{2}$8. Compute $M_{4}={\left[d_{ij}\right]}_{n\times k}$, where $d_{ij}=\frac{|(T-I-F)+\psi |}{2}$.9. Compute $M_{5}=M_{3}⨂M_{4}={\left[e_{ij}\right]}_{m\times k}$.10. Compute scores $S_{i}$ by calculating the row-sum of $M_{5}$.▹**Output:**11. Choose the Max$\left\{S_{i}\right\}$ as final selection.▹**End**

**Example** **3.** ***Input Stage: (Step 1–Step 3)***

*Let six patients be chosen from the Cleveland data set for heart disease diagnosis, and they form the set $U=\{{\hat{p}}_{1},{\hat{p}}_{2},{\hat{p}}_{24},{\hat{p}}_{25},{\hat{p}}_{75},{\hat{p}}_{303}\}$. Let $\hat{D}=\left\{{\hat{D}}_{1},{\hat{D}}_{2},{\hat{D}}_{3},{\hat{D}}_{4}\right\}$ be the set of medical physicians, i.e., decision-makers to assess the diagnosis process, and $E=\{{\hat{e}}_{1},{\hat{e}}_{2},{\hat{e}}_{3},{\hat{e}}_{4},{\hat{e}}_{5},{\hat{e}}_{6},{\hat{e}}_{7},{\hat{e}}_{8},{\hat{e}}_{9}\}$ be the set of attributes with ${\hat{e}}_{1}=$ age, ${\hat{e}}_{2}=$ chest pain type, ${\hat{e}}_{3}=$ resting blood pressure, ${\hat{e}}_{4}=$ serum cholesterol, ${\hat{e}}_{5}=$ fasting blood sugar, ${\hat{e}}_{6}=$ maximum heart rate achieved, ${\hat{e}}_{7}=$ old peak, ${\hat{e}}_{8}=$ slope, and ${\hat{e}}_{9}=$ thal. The attribute-valued disjoint sets corresponding to these attributes are:*

$$E^{1}=\left\{{\hat{e}}^{11}=category1,{\hat{e}}^{12}=category2,{\hat{e}}^{13}=category3,{\hat{e}}^{14}=category4\right\},$$

$$E^{2}=\left\{\begin{array}{c} {\hat{e}}^{21}=typicalangina,{\hat{e}}^{22}=atypicalangina,\\ {\hat{e}}^{23}=non-anginalpain,{\hat{e}}^{24}=asymptomatic \end{array}\right\},$$

$$E^{3}=\left\{{\hat{e}}^{31}=110\;mmHg,{\hat{e}}^{32}=150\;mmHg,{\hat{e}}^{33}=180\;mmHg\right\},$$

$$E^{4}=\left\{{\hat{e}}^{41}=210\;mg/dL,{\hat{e}}^{42}=320\;mg/dL,{\hat{e}}^{43}=430\;mg/dL\right\},$$

$$E^{5}=\left\{{\hat{e}}^{51}=120\;mg/dL\right\},$$

$$E^{6}=\left\{{\hat{e}}^{61}=81,{\hat{e}}^{62}=140\right\},$$

$$E^{7}=\left\{{\hat{e}}^{71}=1.2,{\hat{e}}^{72}=3.7\right\},$$

$$E^{8}=\left\{{\hat{e}}^{81}=up\;sloping,{\hat{e}}^{82}=flat,{\hat{e}}^{83}=down\;sloping\right\},$$

$$E^{9}=\left\{{\hat{e}}^{91}=normal,{\hat{e}}^{92}=fixed\;defect,{\hat{e}}^{93}=reversible\;defect\right\}.$$

***Construction Stage: Step 4:***

*Now, $G=E^{1}\times E^{2}\times E^{3}\times \cdots \times E^{9}=\left\{{\hat{g}}_{1},{\hat{g}}_{2},{\hat{g}}_{3},\ldots ,{\hat{g}}_{r}\right\}$, where r=4×4×3×3×1×2×2×3×3=5184, and each ${\hat{g}}_{i}$ is a nine-tuple element of G.*

***Step 5:***

**Figure 5:**
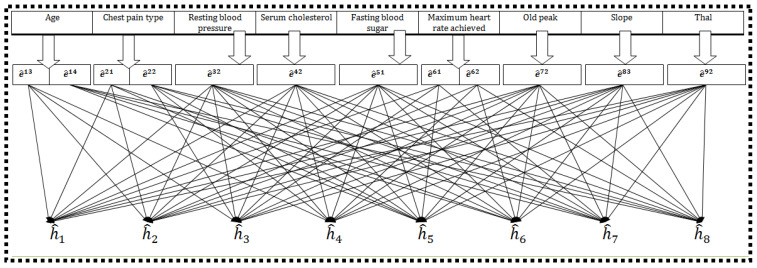
Pictorial representation of the chosen parameters and the determination of their sub-parametric-valued tuples.

*After consultation with the heart specialist, ${\hat{e}}^{13}$ and ${\hat{e}}^{14}$ are preferred in $E^{1}$, ${\hat{e}}^{21}$ and ${\hat{e}}^{22}$ in $E^{2}$, ${\hat{e}}^{32}$ in $E^{3}$, ${\hat{e}}^{42}$ in $E^{4}$, ${\hat{e}}^{51}$ in $E^{5}$, ${\hat{e}}^{61}$, ${\hat{e}}^{62}$ in $E^{6}$, ${\hat{e}}^{72}$ in $E^{7}$, ${\hat{e}}^{83}$ in $E^{8}$, and ${\hat{e}}^{92}$ in $E^{9}$. Therefore, the subset H of G has eight elements, i.e., $H=\{{\hat{h}}_{1},{\hat{h}}_{2},{\hat{h}}_{3},{\hat{h}}_{4},{\hat{h}}_{5},{\hat{h}}_{6},{\hat{h}}_{7},{\hat{h}}_{8}\}$, and each ${\hat{h}}_{i}$ is again a nine-tuple element. Figure 5 explains the Cartesian product of disjoint sets having sub-parametric values corresponding to the chosen attributes. In the first row, the chosen parameters are displayed; their sub-parametric values are presented as disjoint sets below them in the second row, and the last row describes the sub-parametric tuples having multiple arguments.*

***Step 6:***

*Now, two pNHs-sets $A_{\psi }$ and $B_{\zeta }$ are constructed, and their matrix representations $M_{1}={\left[a_{ij}\right]}_{6\times 8}$ and $M_{2}={\left[b_{ij}\right]}_{8\times 4}$ are presented in Table 7 and Table 8, respectively.*

***Computation Stage: Step 7–Step 8:***

*As the entries of matrices $M_{1}$ and $M_{2}$ are in the form of pNHs values, these are transformed into fuzzy values by using the formula $\frac{|(T-I-F)+\psi |}{2}$, and the new matrices $M_{3}$ and $M_{4}$ thus obtained are presented in Table 9 and Table 10, respectively. This transformation of pNHs values to fuzzy values is performed to ensure a discrete decision.*

***Step 9:***

*By using the classical concept of matrix multiplication, the matrix $M_{5}$ of order 6×4 is obtained by multiplying $M_{3}$ and $M_{4}$, which is presented in Table 11. For example, the first row of $M_{3}$ is multiplied by the first column of $M_{4}$, and we obtain 0.295, i.e., 0.295=(0.2×0.15)+(0.1×0.15)+(0.05×0.25)+(0.15×0.1)+(0.4×0.05)+(0.2×0.35)+(0.3×0.4)+(0.25×0.05).*

***Step 10:***

*In order to have an appropriate decision, the score values of patients ${\hat{p}}_{i}$ (under observation) are computed by taking the row-sum in decision matrix $M_{5}$ and are presented in Table 12. For example, the score 1.4675 for ${\hat{p}}_{1}$ is computed by summing up the values in the first row of $M_{5}$, i.e., 1.4675=0.295+0.475+0.325+0.3725.*

***Output Stage: Step 11***

**Figure 6:**
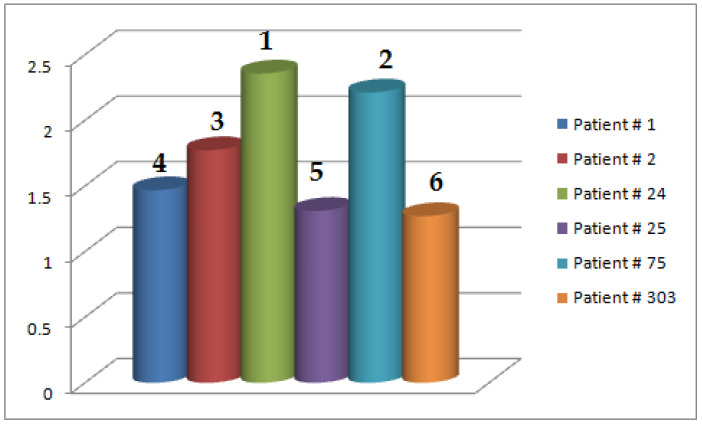
Graphical representation of accumulated score values corresponding to each patient ${\hat{p}}_{i}$ given in Table 12 ( the symbol # denotes for numbering order).

*From Table 12 and Figure 6, it is vividly seen that the patient ${\hat{p}}_{24}$ is more suspected to have heart disease as compared to the others. The ranking of patients for heart disease diagnosis is ${\hat{p}}_{24}>{\hat{p}}_{75}>{\hat{p}}_{2}>{\hat{p}}_{1}>{\hat{p}}_{25}>{\hat{p}}_{303}$.*

**Figure 7:**
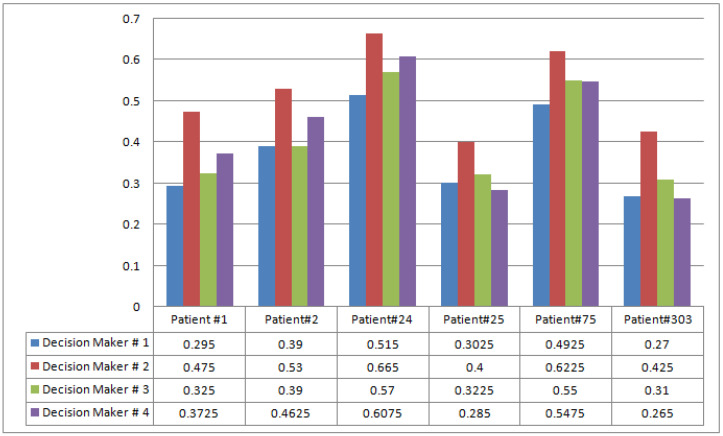
Accumulated decision values of decision-makers corresponding to patients.

The graphical representation of data given in Table 11 is presented in Figure 7 ( the symbol # denotes for numbering order).

## 5. Discussion and Comparison Analysis

In the literature, the medical diagnosis of various diseases has already been discussed by several researchers under neutrosophic set-like models. The data used in these approaches are of a hypothetical nature with general results. On the contrary, the proposed study employed a real data set, the Cleveland data set, to diagnose and analyze the risk of heart diseases. In fact, medical diagnosis is a scenario that requires further partitioning of parameters into their respective sub-parametric values in the form of disjoint sets, which is ignored by existing approaches. Such a kind of classification ensures reliable results and decisions. Meanwhile, in order to reduce the computational complexities, an easy but efficient method, Sanchez’s method, was adopted for this diagnosis. As stated earlier, there are 74 attributes in the Cleveland data set, and it would be a tough task to consider all for the computations; therefore, with the consultation with medical specialists, only the most relevant nine attributes with a key role were chosen. The sub-parametric values with respect to these attributes are also of a real nature, in accordance with the adopted data set. Under this discussion, the main advantages of the proposed study are outlined as:

1. The adopted technique took the idea of parameterization together with the pNHs-set to handle the contemporary decision-making concerns. The supposed possibility degree emulates the uncertain attitude of the level of acknowledgment; in this manner, this has incredible prospects for legitimacy within the domain of computations.
2. Attributes and their sub-attributive values with real values from the Cleveland data set were transformed to fuzzy memberships with an appropriate transformation criterion.
3. As the presented model places emphasis on the comprehensive observation of the parameters and their respective sub-parametric values, it ensures better, reliable, and flexible results from medical physicians as decision-makers.

As the proposed model, the pNHs-set, has not been used previously by any researcher to diagnose heart diseases, the numerical cum computational results of the proposed study cannot be compared with any existing model. However, in order to assess the structural distinction of the proposed model, some characteristics such as the indeterminacy grade (I.G), membership grade (M.G), nonmembership grade (N.M.G), grade of possibility (G.O.P), saa-function (S.A.A.F), and maa-function (M.A.A.F), are considered sufficient for its comparison with the most relevant existing models, such as the FHs-set, IFHs-set, pFs-set, etc. In multi-attribute decision-making, it is often observed that some decision-makers do not want to provide their expert opinions corresponding to the parameters or parametric tuples for the objects under observation and prefer to be neutral in this regard. The inclusion of the I.G ensures such neutrality of the decision-makers. Similarly, the G.O.P ensures that the received expert opinions have the level of acceptance. The omission of these features may lead to a biased decision. This structural comparison is presented in Table 13, which clearly explains that the existing models lack one or more features, but the proposed model satisfies all the features collectively in one model. Hence, it is quite fair to claim that the proposed model is a generalized, flexible, and reliable model compared to the existing ones.

In Table 13, the symbols ✓ and × denote for Yes and No respectively.

## 6. Conclusions

After reviewing the literature, it was observed that the literature has no mathematical model that can tackle the decision-making situations collectively in one model, such as (a) the situation that calls for the necessary categorization of parameters into their related sub-parametric values in the form of different sets, (b) the situation that demands the use of the maa-function, which is capable of tackling the multi-argument domain in the form of sub-parametric-valued tuples, (c) the situation that requires the decision-makers to provide their expert opinions in the form of neutrosophic values, which ensure three dimensions, i.e., truth (real membership), indeterminacy (neutral value), and falsity (real nonmembership), of the opinion of the decision-makers, and (d) the situation that demands the consideration of the possibility degree to assess the acceptance level of the received expert opinions for the objects under consideration. In order to address the limitations of the literature, the novel notions of the pNHs-set were investigated in this study. The aggregations of the pNHs-set were utilized in the medical diagnosis of heart diseases. The input variables, i.e., attributes and sub-attributive values, were taken from the Cleveland data set to evaluate the real prospects of the proposed model. The operational role of each chosen attribute was discussed with a description of their sub-attribute values. The real values of the attributes and their sub-attributive values were transformed to the respective possibility grades by employing suitable mathematical criteria. An integrated algorithm based on the pNHs-set and the modified Sanchez’s method was proposed and then validated by discussing a real-world scenario for the diagnosis of heart diseases. Only nine attributes were considered, and more reliable results were obtained due to the deep focus on evaluating the parameters. Although the proposed study provided a simple, but general framework for medical diagnosis to avoid computational complexity, it can easily be extended as a case study for other uncertain environments such as fuzzy-set-like models with possibility settings by using more attributes and patients from the Cleveland data set. The proposed study has some limitations for managing the situations: (i) the situation that has the information and data regarding the attributes, sub-attribute values, and opinions of decision-makers are in the form intervals, (ii) the situation that has a periodic nature of the information and data, and (iii) the situation that has a rough nature of the information and data. Its scope may cover a wide range of multidisciplinary fields of study such as soft computing, fuzzy logic, artificial intelligence, theoretical computer science, pattern recognition, etc.

## Figures and Tables

**Table 1:** List of abbreviations.

Abbreviation	Stands for	Abbreviation	Stands for	Abbreviation	Stands for
F-set	Fuzzy set	IF-set	Intuitionistic fuzzy set	N-set	Neutrosophic set
S-set	Soft set	Fs-set	Fuzzy soft set	IFs-set	Intuitionistic fuzzy soft set
Ns-set	Neutrosophic soft set	Hs-set	Hypersoft set	maa-function	multi-argument function
FHs-set	Fuzzy hypersoft set	IFHs-set	Intuitionistic fuzzy hypersoft set	NHs-set	Neutrosophic hypersoft set
pFs-set	Possibility fuzzy soft set	pIFs-set	Possibility intuitionistic fuzzy soft set	pNs-set	Possibility neutrosophic soft set
pNHs-set	Possibility neutrosophic hypersoft set	Z	Universal set	P(Z)	Power set of Z
F(Z)	Family of fuzzy sets	IF(Z)	Family of intuitionistic fuzzy sets	N(Z)	Family of neutrosophic sets

**Table 2:** Attributes of the Cleveland data set.

Sr. No.	Sr. No.	Attributes	Attributes
by Analysis	by Data Set	(Abbreviations)	(Full Names)
1	3	age	Age in years
2	4	sex	Sex (male/female)
3	9	cp	Chest pain type)
4	10	trestpbs	Resting blood pressure (mm Hg)
5	12	chol	Serum cholesterol (mg/dL)
6	16	fbs	Fasting blood sugar (120 mg/dL)
7	19	restecg	Resting electrocardiographic results
8	32	Thalach	Maximum heart rate achieved
9	38	Exang	Exercise-induced angina
10	40	Oldpeak	ST depression induced by exercise relative to rest
11	41	slope	The slope of the peak exercise ST segment
12	44	ca	Number of major vessels (0–3) colored by fluoroscopy
13	51	thal	3 = normal; 6 = fixed defect; 7 = reversible defect
14	58	num	Diagnosis of heart disease (angiographic disease status)

**Table 3:** Values corresponding to the selected attributes.

Sr. No.	Sr. No.	Attributes	Attributes	Values Corresponding
by Analysis	by Data Set	(Abbreviations)	(Full Names)	to Attributes in Data Set
1	3	age	Age in years	0–20, 21–40, 41–60, Above 60
3	9	cp	Chest pain type	1. Typical angina, 2. atypical angina, 3. non-anginal pain, 4. asymptomatic
4	10	trestpbs	Resting blood pressure (mm Hg)	90–200mm Hg
5	12	chol	Serum cholesterol (mg/dL)	126–564 mg/dL
6	16	fbs	Fasting blood sugar (120 mg/dL)	120 mg/dL
8	32	Thalach	Maximum heart rate achieved	71–195
10	40	Oldpeak	ST depression induced by exercise relative to rest	0.0–5.6
11	41	slope	The slope of the peak exercise ST segment	1. upsloping, 2. flat, 3. downsloping
13	51	thal	3 = normal; 6 = fixed defect; 7 = reversible defect	1. normal, 2. fixed defect, 3. reversible defect

**Table 4:** Types of cholesterol and their healthy ranges.

Healthy Serum Cholesterol	Less than 200 mg/dL
Healthy HDL Cholesterol	Higher than 55 mg/dL for women and 45 mg/dL for men
Healthy LDL Cholesterol	Less than 130 mg/dL
Healthy Triglycerides	Less than 150 mg/dL

**Table 5:** Ranges of blood sugar.

Blood Sugar (mg/dL)	Interpretation
Above 250	Very high
181–250	High
70–180	In target range
55–69	Low
Below 55	Very low

**Table 6:** Possibility grades corresponding to the selected attributes.

Selected Attributes	Prescribed Values in Data Set	Transformed Fuzzy Values
Age	0–20, 21–40, 41–60, 61–80	0–0.25, 0.2625–0.50, 0.5125–0.75, 0.7625–1.00
Chest pain type)	1, 2, 3, 4	0.25, 0.50, 0.75, 1.00
Resting blood pressure	90–200	0.45–1.00
Serum cholesterol	126–564	0.2234–1.0000
Fasting blood sugar	0, 120	0,1
Maximum heart rate achieved	71–195	0.3641–1.0000
Oldpeak	0.0–5.6	0–1
Slope	1, 2, 3	0.33, 0.66, 1.00
Thal	3, 6, 7	0.4286, 0.8571, 1.0000

**Table 7:** Matrix notation of pNHs-set $A_{\psi }$.

$M_{1}$	${\hat{h}}_{1}$	${\hat{h}}_{2}$	${\hat{h}}_{3}$	${\hat{h}}_{4}$
${\hat{p}}_{1}$	(≺0.5,0.3,0.4≻,0.6)	(≺0.4,0.4,0.3≻,0.5)	(≺0.1,0.2,0.4≻,0.6)	(≺0.2,0.4,0.6≻,0.5)
${\hat{p}}_{2}$	(≺0.1,0.2,0.4≻,0.3)	(≺0.3,0.4,0.1≻,0.6)	(≺0.6,0.3,0.5≻,0.7)	(≺0.6,0.1,0.3≻,0.4)
${\hat{p}}_{24}$	(≺0.6,0.3,0.1≻,0.7)	(≺0.7,0.3,0.2≻,0.6)	(≺0.8,0.1,0.1≻,0.7)	(≺0.3,0.1,0.4≻,0.8)
${\hat{p}}_{25}$	(≺0.6,0.1,0.4≻,0.4)	(≺0.7,0.2,0.3≻,0.8)	(≺0.8,0.4,0.8≻,0.5)	(≺0.4,0.3,0.1≻,0.3)
${\hat{p}}_{75}$	(≺0.9,0.2,0.5≻,0.6)	(≺0.8,0.1,0.4≻,0.7)	(≺0.8,0.3,0.1≻,0.9)	(≺0.6,0.1,0.5≻,0.5)
${\hat{p}}_{303}$	(≺0.3,0.1,0.7≻,0.6)	(≺0.9,0.3,0.5≻,0.7)	(≺0.1,0.4,0.2≻,0.8)	(≺0.9,0.8,0.3≻,0.5)
$M_{1}$	${\hat{h}}_{5}$	${\hat{h}}_{6}$	${\hat{h}}_{7}$	${\hat{h}}_{8}$
${\hat{p}}_{1}$	(≺0.6,0.2,0.1≻,0.5)	(≺0.6,0.2,0.7≻,0.7)	(≺0.6,0.2,0.3≻,0.5)	(≺0.7,0.2,0.4≻,0.4)
${\hat{p}}_{2}$	(≺0.7,0.5,0.1≻,0.3)	(≺0.6,0.2,0.7≻,0.8)	(≺0.9,0.1,0.7≻,0.4)	(≺0.8,0.3,0.3≻,0.4)
${\hat{p}}_{24}$	(≺0.5,0.3,0.4≻,0.6)	(≺0.5,0.3,0.4≻,0.6)	(≺0.5,0.3,0.4≻,0.7)	(≺0.5,0.3,0.4≻,0.8)
${\hat{p}}_{25}$	(≺0.5,0.3,0.4≻,0.7)	(≺0.5,0.3,0.4≻,0.9)	(≺0.5,0.3,0.4≻,0.3)	(≺0.5,0.3,0.4≻,0.5)
${\hat{p}}_{75}$	(≺0.5,0.3,0.4≻,0.6)	(≺0.5,0.3,0.4≻,0.7)	(≺0.5,0.3,0.4≻,0.5)	(≺0.5,0.3,0.4≻,0.7)
${\hat{p}}_{303}$	(≺0.5,0.3,0.4≻,0.8)	(≺0.5,0.3,0.4≻,0.6)	(≺0.5,0.3,0.4≻,0.5)	(≺0.5,0.3,0.4≻,0.4)

**Table 8:** Matrix notation of pNHs-set $B_{\zeta }$.

$M_{2}$	${\hat{D}}_{1}$	${\hat{D}}_{2}$	${\hat{D}}_{3}$	${\hat{D}}_{4}$
${\hat{h}}_{1}$	(≺0.3,0.7,0.5≻,0.6)	(≺0.4,0.6,0.3≻,0.7)	(≺0.5,0.4,0.8≻,0.4)	(≺0.6,0.5,0.7≻,0.3)
${\hat{h}}_{2}$	(≺0.4,0.8,0.6≻,0.7)	(≺0.5,0.7,0.4≻,0.1)	(≺0.6,0.5,0.9≻,0.5)	(≺0.7,0.4,0.6≻,0.4)
${\hat{h}}_{3}$	(≺0.5,0.9,0.7≻,0.6)	(≺0.6,0.8,0.5≻,0.2)	(≺0.7,0.6,0.1≻,0.6)	(≺0.8,0.3,0.5≻,0.5)
${\hat{h}}_{4}$	(≺0.6,0.1,0.8≻,0.5)	(≺0.7,0.9,0.6≻,0.2)	(≺0.8,0.7,0.2≻,0.7)	(≺0.9,0.2,0.4≻,0.6)
${\hat{h}}_{5}$	(≺0.7,0.2,0.9≻,0.3)	(≺0.8,0.1,0.7≻,0.9)	(≺0.9,0.8,0.3≻,0.8)	(≺0.1,0.1,0.3≻,0.7)
${\hat{h}}_{6}$	(≺0.8,0.3,0.1≻,0.3)	(≺0.9,0.2,0.8≻,0.3)	(≺0.1,0.9,0.4≻,0.9)	(≺0.2,0.9,0.2≻,0.8)
${\hat{h}}_{7}$	(≺0.9,0.4,0.2≻,0.5)	(≺0.1,0.3,0.9≻,0.2)	(≺0.2,0.1,0.5≻,0.1)	(≺0.3,0.8,0.1≻,0.9)
${\hat{h}}_{8}$	(≺0.1,0.5,0.3≻,0.6)	(≺0.2,0.4,0.1≻,0.6)	(≺0.3,0.2,0.6≻,0.2)	(≺0.4,0.7,0.9≻,0.1)

**Table 9:** Matrix notation of pNHs-set $A_{\psi }$ after the conversion of the possibility neutrosophic values to reduced fuzzy values.

$M_{3}$	${\hat{h}}_{1}$	${\hat{h}}_{2}$	${\hat{h}}_{3}$	${\hat{h}}_{4}$	${\hat{h}}_{5}$	${\hat{h}}_{6}$	${\hat{h}}_{7}$	${\hat{h}}_{8}$
${\hat{p}}_{1}$	0.2	0.1	0.05	0.15	0.4	0.2	0.3	0.25
${\hat{p}}_{2}$	0.1	0.2	0.25	0.3	0.2	0.25	0.35	0.3
${\hat{p}}_{24}$	0.45	0.4	0.65	0.3	0.2	0.2	0.25	0.3
${\hat{p}}_{25}$	0.25	0.5	0.05	0.15	0.25	0.35	0.05	0.15
${\hat{p}}_{75}$	0.4	0.5	0.65	0.25	0.2	0.25	0.15	0.25
${\hat{p}}_{303}$	0.05	0.4	0.15	0.15	0.3	0.2	0.15	0.1

**Table 10:** Matrix notation of pNHs-set $B_{\zeta }$ after the conversion of the possibility neutrosophic values to reduced fuzzy values.

$M_{4}$	${\hat{D}}_{1}$	${\hat{D}}_{2}$	${\hat{D}}_{3}$	${\hat{D}}_{4}$
${\hat{h}}_{1}$	0.15	0.1	0.15	0.15
${\hat{h}}_{2}$	0.15	0.25	0.15	0.05
${\hat{h}}_{3}$	0.25	0.25	0.3	0.25
${\hat{h}}_{4}$	0.1	0.3	0.3	0.35
${\hat{h}}_{5}$	0.05	0.45	0.3	0.2
${\hat{h}}_{6}$	0.35	0.1	0.15	0.05
${\hat{h}}_{7}$	0.4	0.45	0.15	0.15
${\hat{h}}_{8}$	0.05	0.15	0.1	0.55

**Table 11:** Matrix notation of $M_{5}=M_{3}⨂M_{4}$.

$M_{5}$	${\hat{D}}_{1}$	${\hat{D}}_{2}$	${\hat{D}}_{3}$	${\hat{D}}_{4}$
${\hat{p}}_{1}$	0.295	0.475	0.325	0.3725
${\hat{p}}_{2}$	0.39	0.53	0.39	0.4625
${\hat{p}}_{24}$	0.515	0.665	0.57	0.6075
${\hat{p}}_{25}$	0.3025	0.4	0.3225	0.285
${\hat{p}}_{75}$	0.4925	0.6225	0.55	0.5475
${\hat{p}}_{303}$	0.27	0.425	0.31	0.265

**Table 12:** Score values $S({\hat{p}}_{i})$ corresponding to each patient ${\hat{p}}_{i}$.

${\hat{p}}_{i}$	$S({\hat{p}}_{i})$
${\hat{p}}_{1}$	1.4675
${\hat{p}}_{2}$	1.7725
${\hat{p}}_{24}$	2.3575
${\hat{p}}_{25}$	1.3100
${\hat{p}}_{75}$	2.2125
${\hat{p}}_{303}$	1.2700

**Table 13:** Advantageous aspects of the proposed approach.

Authors	Structure	I.G	M.G	N.M.G	G.O.P	S.A.A.F	M.A.A.F
Debnath [15]	FHs-set	×	✓	×	×	✓	✓
Yolcu et al. [16]	IFHs-set	×	✓	✓	×	✓	✓
Alkhazaleh et al. [24]	pFs-set	×	✓	×	✓	✓	×
Bashir et al. [25]	pIFs-set	×	✓	✓	✓	✓	×
Karaaslan [26]	pNs-set	✓	✓	✓	✓	✓	×
Rahman et al. [29]	pIFHs-set	×	✓	✓	✓	✓	✓
Proposed Model	pNHs-set	✓	✓	✓	✓	✓	✓

## Data Availability

In this research, the data relating to attributes and their sub-attributes are taken from the Cleveland Data set (heart disease data set) which is freely available online at (http://archive.ics.uci.edu/ml/datasets/Heart+Disease) (accessed on 3 October 2021).

## References

[1] Zadeh L.A. (1965). Fuzzy sets. Inf. Control.

[2] Atanassov K. (1986). Intuitionistic fuzzy sets. Fuzzy Sets Syst..

[3] Smarandache F. (1998). Neutrosophy, Neutrosophic Probability, Set, and Logic, Analytic Synthesis and Synthetic Analysis.

[4] Molodtsov D. (1999). Soft set theory—First results. Comput. Math. Appl..

[5] Maji P., Biswas R., Roy A. (2003). Soft set theory. Comput. Math. Appl..

[6] Ali M.I., Feng F., Liu X., Min W.K., Shabir M. (2009). On some new operations in soft set theory. Comput. Math. Appl..

[7] Maji P.K., Biswas R., Roy A.R. (2001). Fuzzy soft sets. J. Fuzzy Math..

[8] Maji P.K., Biswas R., Roy A.R. (2001). Intuitionistic fuzzy soft sets. J. Fuzzy Math..

[9] Maji P.K. (2013). Neutrosophic soft set. Ann. Fuzzy Math. Inform..

[10] Smarandache F. (2018). Extension of soft set of hypersoft set, and then to plithogenic hypersoft set. Neutrosophic Sets Syst..

[11] Abbas F., Murtaza G., Smarandache F. (2020). Basic operations on hypersoft sets and hypersoft points. Neutrosophic Sets Syst..

[12] Saeed M., Rahman A.U., Ahsan M., Smarandache F. (2021). An inclusive study on fundamentals of hypersoft set. Theory and Application of Hypersoft Set.

[13] Rahman A.U., Saeed M., Smarandache F., Ahmad M.R. (2020). Development of hybrids of hypersoft set with complex fuzzy set, complex intuitionistic fuzzy set and complex neutrosophic set. Neutrosophic Sets Syst..

[14] Rahman A.U., Saeed M., Smarandache F. (2020). Convex and concave hypersoft sets with some properties. Neutrosophic Sets Syst..

[15] Debnath S. (2021). Fuzzy hypersoft sets and its weightage operator for decision making. J. Fuzzy Ext. Appl..

[16] Yolcu A., Smarandache F., Öztürk T.Y. (2021). Intuitionistic fuzzy hypersoft sets. Commun. Fac. Sci. Univ. Ank. Ser. Math. Stat..

[17] Saqlain M., Saeed M., Ahmad M.R., Smarandache F. (2020). Generalization of TOPSIS for neutrosophic hypersoft sets using accuracy function and its application. Neutrosophic Sets Syst..

[18] Saeed M., Ahsan M., Saeed M.H., Mehmood A., Abdeljawad T. (2021). An application of neutrosophic hypersoft mapping to diagnose hepatitis and propose appropriate treatment. IEEE Access.

[19] Saeed M., Ahsan M., Rahman A.U., Mehmood A. (2021). An application of neutrosophic hypersoft mapping to diagnose brain tumor and propose appropriate treatment. J. Intell. Fuzzy Syst..

[20] Saeed M., Rahman A.U., Arshad M., Dhital A. (2021). A novel approach to neutrosophic hypersoft graphs with properties. Neutrosophic Sets Syst..

[21] Saeed M., Rahman A.U., Arshad M. (2021). A study on some operations and products of neutrosophic hypersoft graphs. J. Appl. Math. Comput..

[22] Rahman A.U., Saeed M., Dhital A. (2021). Decision making application based on neutrosophic parameterized hypersoft set theory. Neutrosophic Sets Syst..

[23] Rahman A.U., Saeed M., Alodhaibi S.S., Khalifa H.A.E.W. (2021). Decision making algorithmic approaches based on parameterization of neutrosophic set under hypersoft set environment with fuzzy, intuitionistic fuzzy and neutrosophic settings. CMES Comput. Model. Eng. Sci..

[24] Alkhazaleh S., Salleh A.R., Hassan N. (2011). Possibility fuzzy soft set. Adv. Decis. Sci..

[25] Bashir M., Salleh A.R., Alkhazaleh S. (2012). Possibility intuitionistic fuzzy soft set. Adv. Decis. Sci..

[26] Karaaslan F. (2016). Similarity measure between possibility neutrosophic soft sets and its applications. Univ. Politeh. Buchar. Sci. Bull. Ser. A Appl. Math. Phys..

[27] Sanchez E. (1979). Inverses of fuzzy relations. Application to possibility distributions and medical diagnosis. Fuzzy Sets Syst..

[28] (2010). UC Irvine Machine Learning Repository, Cleveland Heart Disease Data Details. Dataset. https://archive.ics.uci.edu/ml/datasets/heart+Disease.

[29] Rahman A.U., Saeed M., Khalifa H.A.E.-W., Afifi W.A. (2022). Decision making algorithmic techniques based on aggregation operations and similarity measures of possibility intuitionistic fuzzy hypersoft sets. AIMS Math..

